# Multimodal characterization of sustained bioagent release from an epicardial depot for long-term biomaterial incorporation

**DOI:** 10.1016/j.biomaterials.2026.124087

**Published:** 2026-02-18

**Authors:** Claudia E. Varela, David S. Monahan, Yiling Fan, Shahrin Islam, Jane Tunde Kelleher, William Whyte, Jean Bonnemain, Souen Ngoy, Sudeshna Fisch, Eimear Wallace, William Ronan, Rachel Beatty, Eimear B. Dolan, Christopher T. Nguyen, Garry P. Duffy, Ellen T. Roche

**Affiliations:** a Institute for Medical Engineering and Science, Massachusetts Institute of Technology, Cambridge, MA, USA; b Harvard-MIT Program in Health Sciences and Technology, Cambridge, MA, USA; c CÚRAM Research Ireland Centre for Medical Devices, College of Medicine Nursing and Health Sciences, University of Galway, Galway, Ireland; d Department of Mechanical Engineering, Massachusetts Institute of Technology, Cambridge, MA, USA; e Department of Adult Intensive Care Medicine, Lausanne University Hospital and University of Lausanne, Lausanne, Switzerland; f Department of Medicine, Brigham & Women’s Hospital and Harvard Medical School, Boston, MA, USA; g Biomedical Engineering, College of Engineering and Science, University of Galway, Ireland; h Anatomy Department, College of Medicine, Nursing and Health Sciences, University of Galway, Ireland; i Cardiovascular Institute, Cleveland Clinic, OH, USA; j Anatomy and Regenerative Medicine, Royal College of Surgeons in Ireland, 123 St. Stephen’s Green, Dublin, Ireland

**Keywords:** Multidose, Epicardial drug delivery, Transport modeling, Myocardial infarction, Rat model

## Abstract

Epicardial delivery of therapies has the potential to prevent adverse remodeling and promote *in situ* regeneration after myocardial infarction (MI) but further optimization of bioagent dosing and transport to heart muscle is required to maximize their therapeutic potential. Replenishable reservoir systems have enabled localized bioagent delivery to the epicardial surface but therapy transport from these systems is constrained by semi-permeable membranes and fibrous capsule formation. Our approach to improved therapy delivery from epicardial reservoir systems is multi-pronged. First, we introduce a membrane-free reservoir system by incorporating a gelatin scaffold into a flexible polymer implant to promote direct integration with the epicardial surface and act as a replenishable depot to encourage myocardial-directed transport. Next, we perform in vitro and *ex vivo* validations and multi-scale computational simulations to characterize biomaterial, tissue, and organ-level transport of therapy, considering both native tissue architecture, and the effect of blood vessel clearance. As an *in vivo* use case of our system, we investigated the functional effect of multi-dose regimens of human follistatin-like 1 protein (FSTL1) in a rat model of myocardial infarction (MI). Groups receiving multiple doses of FSTL1 show increased cardiac performance (ejection fraction and fractional shortening), and decreased chamber stiffness 28 days after MI. Multi-dosing increases ventricular wall thickness and reduces infarct size. We demonstrate a dose-dependent increase in blood vessel number and density in the infarct zone. Finally, we establish a computational and experimental framework for patient-specific modeling to optimize implant parameters such as reservoir size and shape, infarct location, and dosing regimens, with a vision for clinical-imaging guided bioagent delivery strategies that can be modified on a per-patient, therapy-specific basis to optimize dosing regimens of various bioagents. This study highlights the potential for integrating personalized computational models with replenishable delivery systems to improve bioagent transport from biomaterials and enhance post-MI therapeutic outcomes.

## 1. Introduction

A myocardial infarction (MI), or heart attack, occurs when blood flow to a region of myocardium is interrupted, leading to an infarcted area that can undergo adverse tissue remodeling. Ultimately, this remodeling can lead to heart failure (HF)—a complex syndrome where the heart cannot pump enough blood to meet the body’s metabolic demands. Despite current clinical management strategies, HF remains a prominent cause of morbidity and mortality in MI survivors [1], highlighting the clinical need for therapeutic strategies that promote early recovery of cardiac muscle and function after MI. Pre-clinical evidence indicates that delivering bioagents, such as stem cells, growth factors, proteins, and drugs, directly to the infarcted heart can lead to early improvements in cardiac recovery [2–15]. In most studies, these benefits are observed after a single dose of bioagent is delivered by intra-myocardial injection or through a biomaterial carrier coupled to the epicardial heart surface at the time of surgical MI creation [2–15]. Such approaches include hydrogel patches and injections, which serve as depots for therapeutic delivery, and poly (lactic-co-glycolic acid) microspheres that are injected into the myocardium for local, longitudinal release of therapeutic [16–18]. While promising, biomaterial carriers and scaffolds require one-time surgical placement without opportunity to adjust dosing based on patient’s response to therapy. Similarly, while injectables facilitate additional dosing, the process is invasive and drives local inflammation. Replenishable epicardial reservoir systems [19–21] have enabled the non-invasive, localized delivery of multiple bioagent doses to the epicardial surface of the heart. Sustained therapy delivery through these systems is enabled by a bioagent-laden biomaterial depot that is connected to a subcutaneous port via a catheter line for trans-cutaneous replenishment.

Despite these advancements, limitations in replenishable epicardial reservoir systems remain. Firstly, work on epicardial reservoir systems, including an epicardial reservoir with a semi-permeable membrane for targeted therapy delivery developed in our group, demonstrated that bioagent diffusion is significantly limited (~1.7 times slower) by the semipermeable membrane that separates the bioagent-laden biomaterial from the host epicardium and the formation of a fibrous capsule at the membrane/tissue interface [19,22]. Computational studies from our group suggest that removing the membrane at the interface between the bioagent-laden biomaterial and the epicardial surface would amend this, yielding greater penetration of bioagent into the tissue [22]. Moreover, removal of the membrane interfacing with tissue is expected to reduce fibrous capsule formation and allow for integration between the bioagent-laden biomaterial and the host’s epicardium to allow for bidirectional cell migration or mechanical coupling, both of which are beneficial for biological and mechanical therapies.

Although existing epicardial delivery systems allow for preclinical dosing regimen studies of promising bioagents, multiple parameters can be considered to further optimize bioagent transport from these systems, likely leading to improved cardiac benefit *in vivo*. Among these are implant-related parameters such as composition of the implant/tissue interface and implant size, shape, and placement, as well as local cardiac tissue properties like myocyte orientation and degree of vascularity which will change over time based on patient MI location and remodeling stage. To investigate how this type of patient-specific information influences bioagent transport, clinical imaging-guided computational models must be developed and ideally coupled to biological characterizations of sustained bioagent delivery.

In this work, we introduce a replenishable epicardial reservoir system that allows for non-invasive multi-dosing, and direct contact and integration between the bioagent-laden biomaterial and the host’s epicardium to circumvent the rate-limiting nature of porous membranes and the formation of a fibrous capsule at the interface. We select a gelatin sponge as our biomaterial carrier, given its clinical precedence and ability to act as a depot and demonstrate that multiple doses of a bioagent proxy yield better bioagent penetration into *ex vivo* muscle tissue. We perform MRI-guided finite element (FE) simulations of tissue and organ scale transport of a bioagent proxy delivered to the epicardium and model bioagent diffusion patterns in anisotropic tissue with high fidelity to MRI data. Moreover, in a patient-specific left ventricle (LV) model of therapy delivered to the epicardium, we observe that the inclusion of vascular clearance reduces the transmural and surface penetration of therapy, further motivating the need for multiple-dosing to maintain therapeutic bioagent concentrations. As a case study, we use the pro-integration replenishable reservoir platform to characterize the impact of a single, double, and triple dose regimen of FSTL1, a protein known to induce *in situ* cardiomyocyte regeneration for which bidirectional cell migration and biomaterial-epicardial coupling is desirable, on cardiac function, scarring, and angiogenesis in a rat model of MI. Finally, we demonstrate how this computer-aided design approach for epicardial therapies might be expanded to patient-specific modeling to optimize implant size, orientation, infarct size, vascularity, and number of refills. We propose a clinical vision that allows patient imaging and patient-specific modeling to inform epicardial system design, dose regimen, and ultimately, therapeutic outcomes.

## 2. Results and discussion

### 2.1. Design considerations for an epicardial system

**Fig. 1.**
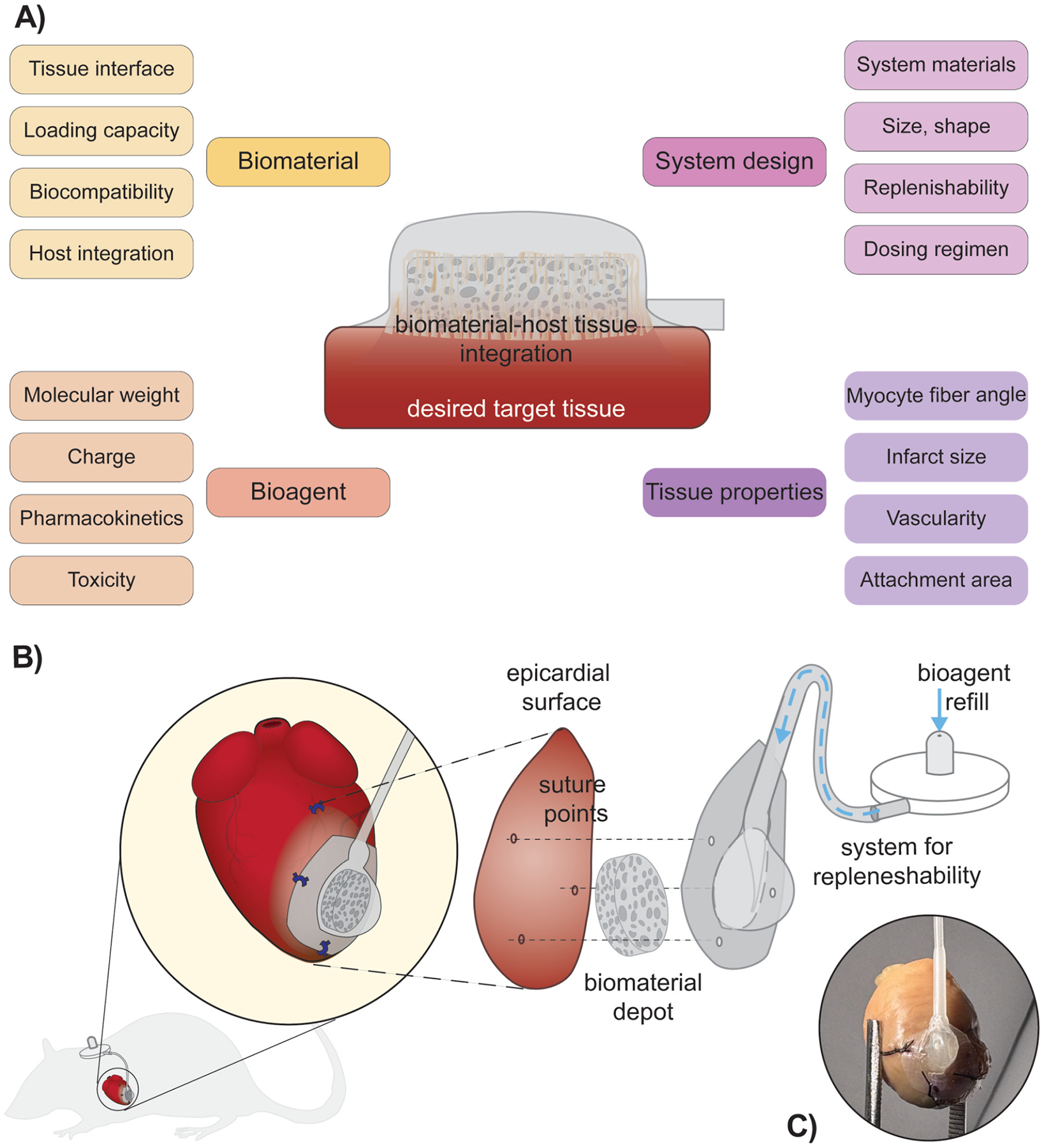
Overview of system design. **A)** Design considerations to enhance bioagent transport from an epicardial reservoir system that allows for biomaterial-host tissue integration. **B)** Schematic of implanted biomaterial-based epicardial reservoir (exploded view) in a rat and trajectory of dose refill after subcutaneous port injection. **C)** Photograph of the epicardial reservoir on a rodent heart.

**Fig. 2.**
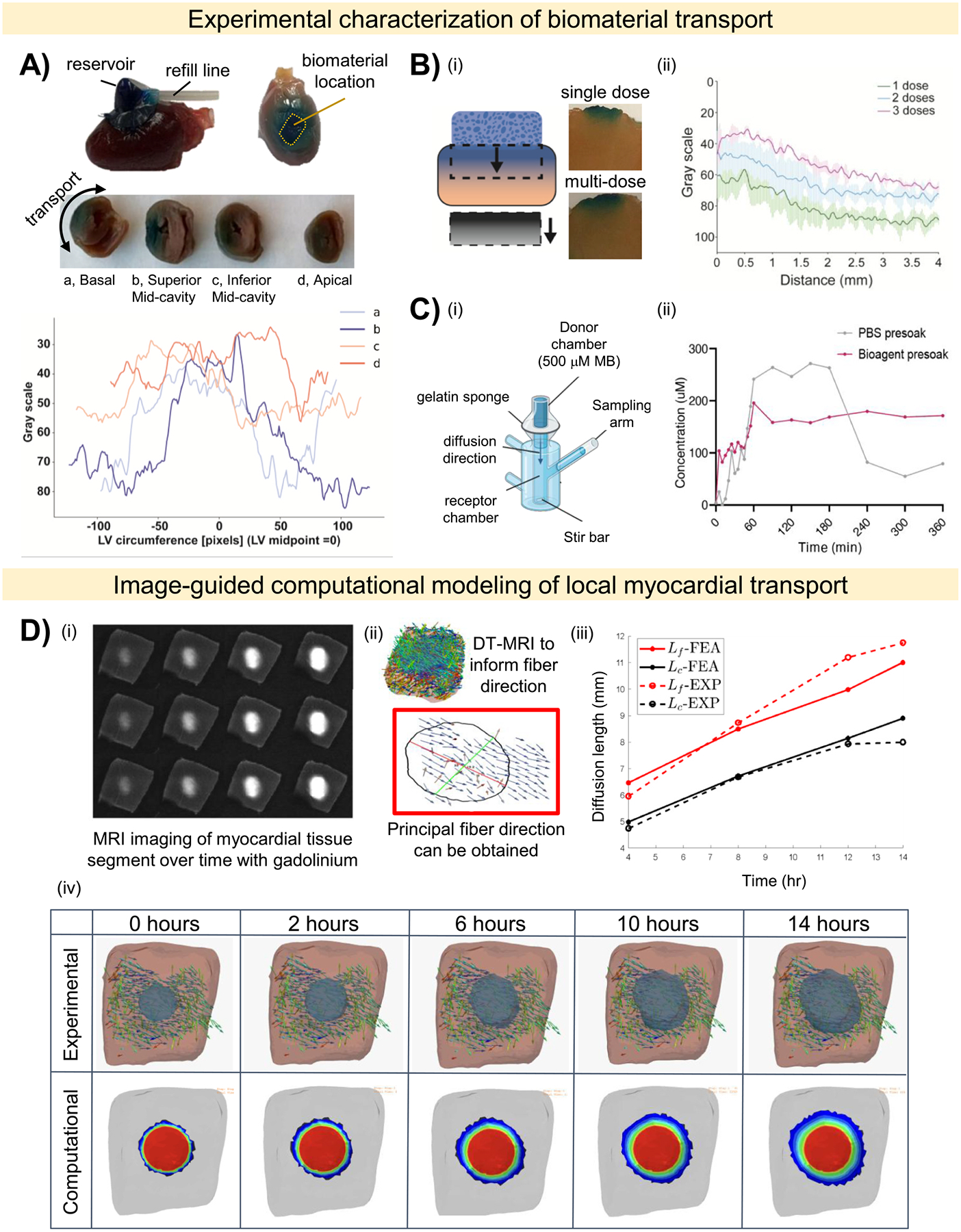
Experimental and computational modeling of bioagent transport from gelatin biomaterial into tissue. **A)** Image of *ex vivo* diffusion experiment on an explanted rat heart. Epicardial and myocardial delivery of bioagent proxy (methylene blue) is evident. Graph of pixel intensity along the surface of the muscle illustrating the spatial profile of bioagent transport with respect to device placement. **B)** (i) The set-up for *ex vivo* demonstration of cumulative drug release with single and multi-dosing regimes. (ii) The graph shows grayscale intensity analysis of the drug analog in tissue orthogonal to the mid-point of the scaffold. **C)** (i) Experimental set up for *in vitro* analysis with a Franz Cell. (ii) The graph demonstrates more consistent delivery with pre-loaded bioagent analog. **D)** (i) MRI imaging with gadolinium-based contrast agent (Gd) (ii) Principal fiber orientation can be derived from diffusion tensor (DT)-MRI imaging. **E)** Diffusion length as predicted by finite element analysis (Computational) correlates well with experimental data as depicted in (iv) for 0–14 h.

Various design features can be considered to enhance bioagent transport from epicardial reservoir systems, potentially resulting in greater therapeutic benefits for the infarcted heart (Fig. 1A). We first remove transport limitations imparted by the presence of semi-permeable membranes between the bioagent-laden biomaterial and the host’s epicardium, allowing for biomaterial-host tissue integration (Fig. 1A and B). A hemispherical polymer reservoir was assembled around a gelatin biomaterial directly interfacing with the epicardial tissue. A refill line connects the hemispherical reservoir to a trans-cutaneous port (Fig. 1B). *Ex vivo* transport experiments were conducted using a bioagent proxy (methylene blue) to assess transport from the proposed membrane-free reservoir system and the selected biomaterial carrier. A reservoir containing a gelatin sponge loaded with one dose of methylene blue was sutured onto the epicardium of an explanted rat heart and allowed to diffuse for 24 h at physiological temperature, after which cross-sections of the tissue were imaged (Fig. 2A). Diffusion of the bioagent was evident at the epicardial surface and within the myocardium (Fig. 2A). The pixel intensity was quantified as a surrogate for drug concentration and illustrates the radial transport profile with respect to the reservoir’s placement on the heart (around the circumference of the LV. In a second *ex vivo* experiment, methylene blue was delivered to muscle tissue with a single, double and triple dose, and the cross-section of the tissue was imaged. Increased penetration of the bioagent through the depth of the tissue increased with the number of doses (Fig. 2B (i,ii)). A Franz Cell study was then conducted with the gelatin biomaterial, demonstrating that pre-soaking the gelatin biomaterial in the bioagent being delivered enabled more consistent delivery of bioagent when compared to pre-soaking with phosphate buffered saline (PBS) over early timepoints. This pre-loading provides a consistent depot of bioagent to ensure optimal transport into myocardial tissue (Fig. 2C (i,ii)), and the depot can be refilled over time (Fig. S1).

### 2.2. Computational simulations of therapy delivery from a reservoir system

#### 2.2.1. Image-guided computational modeling of tissue transport

To characterize diffusion in myocardium, we used T1-mapping MRI to track diffusion of a gadolinium-based contrast agent, gadoterate meglumine (Gd) (Dotarem), into a porcine left ventricular tissue block over 14 h (Fig. 2D(i)), and diffusion tensor MRI to estimate local fiber orientation (Fig. 2D(ii)). Anisotropic diffusivities were extracted from the measured Gd diffusion profile and fiber architecture and used to construct an FE simulation of anisotropic diffusion. The simulated diffusion profiles were thresholded using a concentration cutoff corresponding to the MRI detection limit (0.003 M), and closely resembled elliptical contours aligned with epicardial fiber directions, consistent with a diffusivity ratio of D_s_:D_f_ = 0.58. The FE results closely matched the experimental diffusion profiles, with a slight overprediction of diffusion lengths in both fiber and sheetlet directions (Fig. 2D(iii–iv)).

#### 2.2.2. Image-guided computational modeling of organ scale transport

**Fig. 3.**
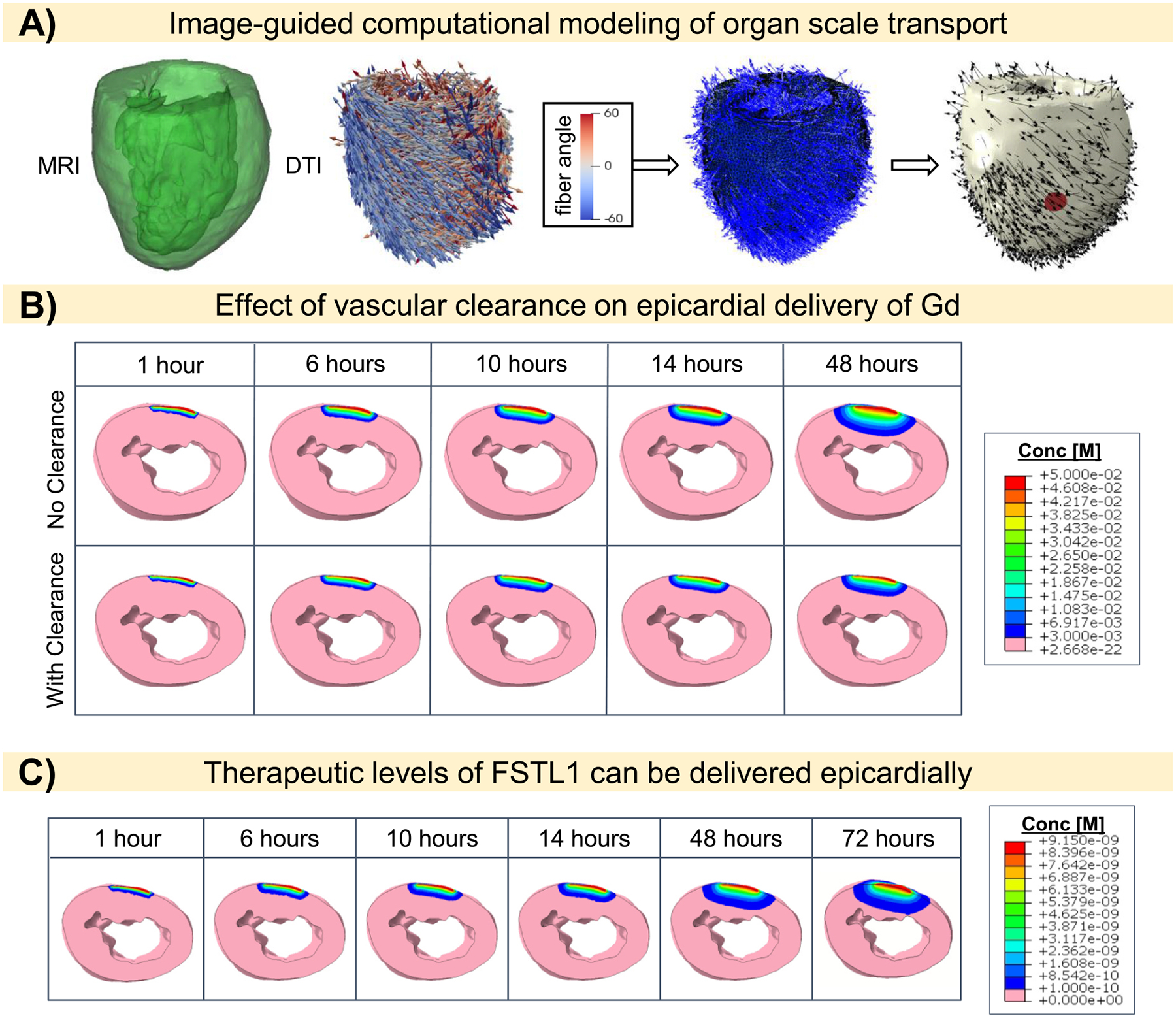
Computational modeling of organ scale bioagent transport. **A)** Magnetic resonance imaging (MRI) and diffusion tensor imaging (DTI) were used to create a patient-specific cardiac model, incorporating myocardial fiber orientation. **B)** The effect of vascular clearance on penetration of a bioagent proxy (Gd) transmurally into the myocardium. **C)** Simulation showing that FSTL1 can reach therapeutic levels in the myocardium through epicardial delivery.

The diffusivity values for the LV were then implemented in a myocardial diffusion model on a patient-specific LV, incorporating fiber orientations derived from diffusion tensor MRI. This model enables virtual testing of various reservoir designs to study their effects on anisotropic diffusion profiles, Fig. 3A. In myocardial tissue, therapeutic agents travel through the extracellular matrix (ECM) by diffusion and are removed upon reaching nearby vasculature, where they are transported into systemic circulation. To assess the impact of vascular clearance on therapy residence, we incorporated a particle size-dependent clearance rate into the diffusion model. Compared to the diffusion-only case, the inclusion of clearance led to a markedly shorter residence time, with the distribution profile reaching steady state within 10 h, Fig. 3B. Penetration depth was also reduced by approximately 50% (from 7.0 mm to 3.6 mm) at the same time point, highlighting the significant effect of microvascular clearance on limiting tissue retention of small molecules like Gd, Fig. 3B.

### 2.3. Preclinical implementation of epicardial system

#### 2.3.1. Multidose FSTL1 regimes lead to improved cardiac function

Previous work on epicardial therapy delivery has leveraged bioactive factors that promote regeneration and angiogenesis and reduce scarring. Candidates with strong evidence in preclinical studies include vascular endothelial growth factor, paracrine factors secreted by progenitor cells, and FSTL1, all of which were found to promote myocardial recovery and angiogenesis while reducing fibrosis. Amongst these, FSTL1 was selected as a model therapy to evaluate the preclinical usability of our platform to improve cardiac recovery after MI with multiple dosing. FSTL1, a glycoprotein that is constitutively expressed in the epicardium and myocardium, regulates cardiac homeostasis and has decreased expression in the epicardium after MI [23]. Prior studies in porcine and mouse MI models have demonstrated that one-dose epicardial repletion of FSTL1 reduces scarring and enhances myocardial regeneration and angiogenesis [23]. Moreover, prior *in vitro* studies have demonstrated that benefits exerted by FSTL1 are dose-dependent [24]. However, repeated FSTL1 delivery via mRNA injection to the heart failed to provide cardiac benefit, potentially due to factors associated with the secondary surgical procedure used to deliver additional doses, such as inflammation and physiological injury to the heart [25]. Therefore, FSTL1 was selected as a prototypical therapy to evaluate clinical usability of our platform because of its demonstrated efficacy in preclinical models, its specificity to the epicardium, and the outstanding need for a method to decouple the therapeutic benefit of repeated FSTL1 dosing from the adverse effects of additional invasive interventions.

To investigate the implications of anisotropic diffusivity and microvascular clearance on epicardial delivery of FSTL1, we simulated FSTL1 diffusion from an epicardial patch into the same patient-specific ventricle from Section 2.2.1. As with Gd, the results (Fig. 3B) demonstrated that microvascular clearance influenced diffusion into the myocardium. Furthermore, with microvascular clearance, the simulation indicated that application of 9.15e-9 M FSTL1 to a 10 mm patch on the epicardium resulted in a concentration varying from the reservoir concentration (9.15e-9 M) to approximately 1e-10 M at the deep penetration of approximately 20 mm into the myocardium after 72 h. Of note, these local concentrations are concordant with FSTL1 doses found to have therapeutic benefit in previous work [23]. These findings support the design of the epicardial FSTL1 depot developed herein, with size adapted for application to the rat heart. To assess the capacity for FSTL1 delivery from the gelatin sponge in our reservoir system, the amount of FSTL1 delivered over 7, 14 and 21 h when additional doses are supplied at 7 and 14 h was quantified using a Franz Cell set up. A linear increase in FSTL1 was measured as the number of doses replenished increased (Fig. S1).

**Fig. 4.**
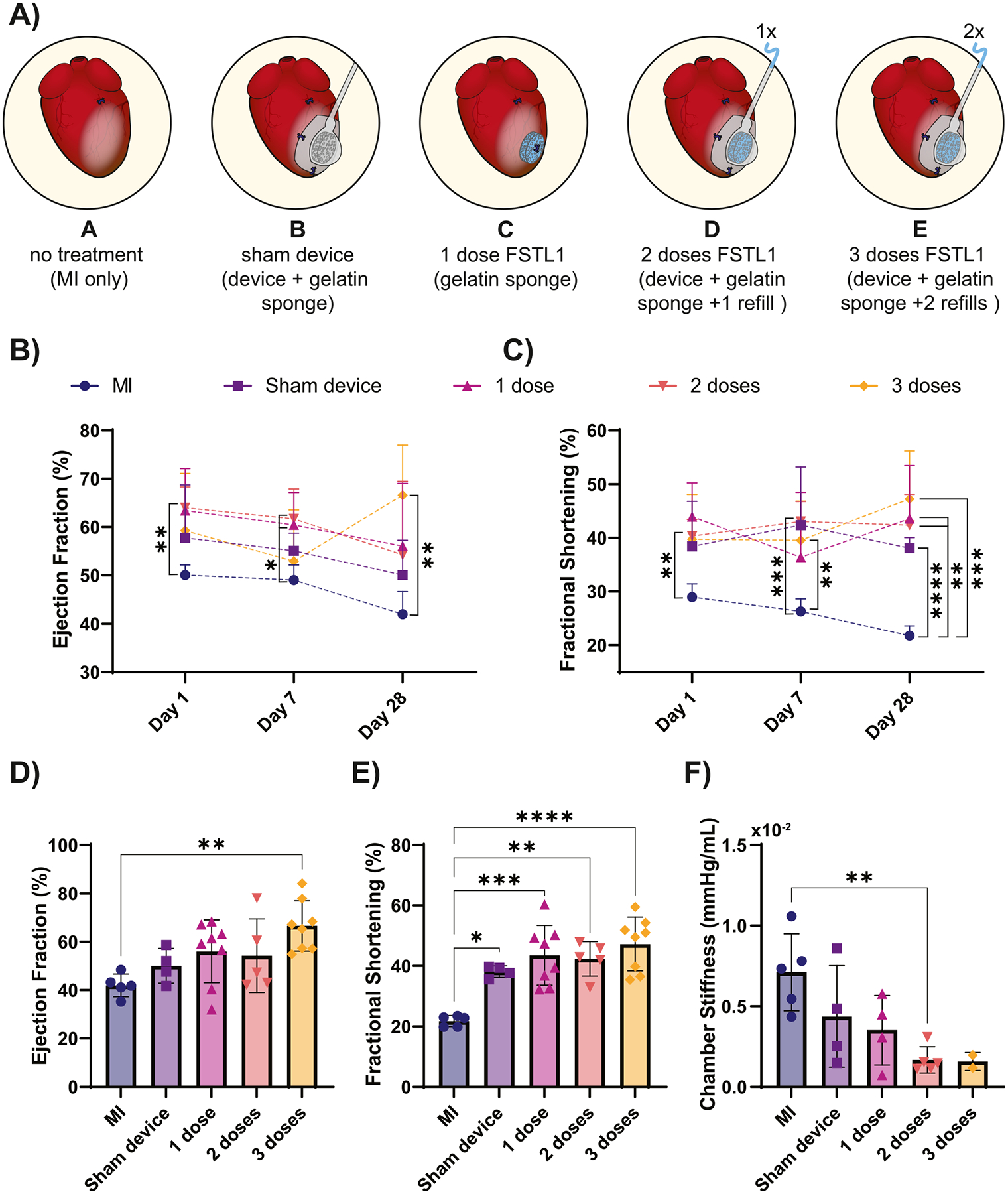
Animal study overview and cardiac function assessment with multiple FSTL1 dose regimens. **A)** Description of animal study groups. Ejection fraction (EF) **(B)** and fractional shortening (FS) **(C)** for all groups at all study timepoints as assessed by echocardiography. Ejection fraction **(D)** and fractional shortening **(E)** at day 28 post-MI for all groups as assessed by echocardiography. **F)** Chamber stiffness at day 28 post-MI for all groups obtained from hemodynamic measurements. *P < 0.05, **P < 0.005, ***P < 0.0005, ****P < 0.0001. Data are mean ± s.d. (n = 2–8) as analyzed by a one-way ANOVA (Mixed model) with Tukey’s multiple comparisons post-test. Individual animal values are shown as points in C-E.

Next, we conducted an FSTL1 dosing study in a rat model of MI of permanent left anterior descending (LAD) coronary artery ligation using our pro-integration reservoir. The study groups are depicted in Fig. 4A. We compared how left ventricular ejection fraction (EF) (Fig. 4B–Fig. S2A) and fractional shortening (FS) (Fig. 4C–Fig. S2B), both echocardiography-derived cardiac function metrics, were impacted by the single, double, and triple FSTL1 dosing regimens over the course of the 28-day study. By day 7 after MI, FSTL1-treated animals had significantly better cardiac function than the MI-only group after only one dose of FSTL1 had been delivered. Specifically, the single and double dose groups had higher EF (Fig. 4B) and the double and triple dose groups had higher FS (Fig. 4C) when compared to the MI only group. By day 28 after MI, only animals treated with 3 doses of FSTL1 had improved EF (Fig. 4B–D, Fig. S2A) when compared to untreated controls. Although the FSTL1-treated and sham implant groups had better FS than MI only animals at day 28, 3 doses of FSTL1 seem to yield the most FS improvement (Fig. 4C–E, Fig. S2B).

By performing invasive hemodynamic measurements in a subset of animals at 28 days post-MI, we show that left ventricular chamber stiffness decreases with FSTL1 delivery in a dose-dependent manner (Fig. 4F). An FSTL1-driven reduction in infarct size and subsequent smaller scar region could explain this decrease in chamber stiffness as well as the recovery in EF and FS in the FSTL1-treated animals when compared to untreated controls. Importantly, chamber stiffness is a parameter derived from the End Diastolic Pressure Volume Relationship (EDPVR) which reflects both myocardial mechanical properties and LV chamber geometry [26]. Changes in EDPVR-derived parameters could arise from changes in myocardial properties or changes in LV geometry. Since we observed that the end diastolic volume (EDV, an indicator of LV geometry) did not significantly vary between our study groups (Fig. S3), the improvement in chamber compliance we report could be attributed to reducing the region of the LV that stiffens due to scar formation through previously reported FSTL1-driven processes [27].

Our results indicate that implantation of our epicardial system led to similar functional changes in the FSTL1-treated and sham implant groups. In the absence of FSTL1, the functional changes in the sham implant group likely arise from the mechanical benefit that implantation of the implant itself has on the infarcted heart. Coupling biologic-free implants and/or biomaterials to the epicardial surface after MI can lead to functional benefits by modulating the biomechanics of the infarcted LV through mechanical restraint and stress transfer to improve pump function or by limiting the degree of LV dilation and functional decrease over the course of MI remodeling [19,28–30]. Our system provides mechanical ventricular support and limiting the reduction in EF and FS over the course of post-MI remodeling by limiting the maximal degree of LV dilation that can occur.

We did not detect significant differences between the dosing regimens in terms of EF, FS or chamber stiffness observed among the single, double, and triple dose regimens even though all functional parameters were better than the no-treatment group. Notably, significant differences were observed between our untreated control and the rest of our experimental groups at day 1, indicating that the coronary ligations generated non-uniform degrees of functional decline. Some factors potentially introducing variability into EF and FS quantifications include variations in MI size/location despite the consistent position of LAD ligation (LV akinesis or thinning is not always visible at the midpapillary plane used to calculate FS) and diminished rat echogenicity based on animal-specific anatomical features. Although a mouse model has been used for other FSTL1 studies, and may have been more easily imaged, miniaturization of the epicardial system to mouse size is challenging. A limitation of this study is that invasive hemodynamic recordings were only performed in a subset of the animals. Future studies could further elucidate the physiological impact of multidose FSTL1 regimes by mitigating these sources of variability and limitations through MRI-based quantifications of cardiac function and biomechanics (e.g. strain). Monitoring clinically relevant biomarkers could allow for clustering of animals based on disease severity or treatment response and further elucidate if additional FSTL1 doses lead to a cumulative functional impact in certain disease conditions or are simply limited in our chosen model.

#### 2.3.2. Multidose FSTL1 regimes lead to improved healing, remodeling, and angiogenesis

To further assess the FSTL1 cardiac benefits that may not be fully captured with echo-based functional metrics, we histologically evaluated the impact of FSTL1 in infarct size, ventricular thickness, and angiogenesis in an arbitrary subset of excised heart samples. An assessment of the foreign body response elicited by the implanted system using the sham device samples indicated hallmarks of late granulation tissue formation surrounding the reservoir (Fig. S4), indicative of a chronic inflammatory response that is comparable to previous reports [19].

##### 2.3.2.1. Infarct size and LV thickness.

**Fig. 5.**
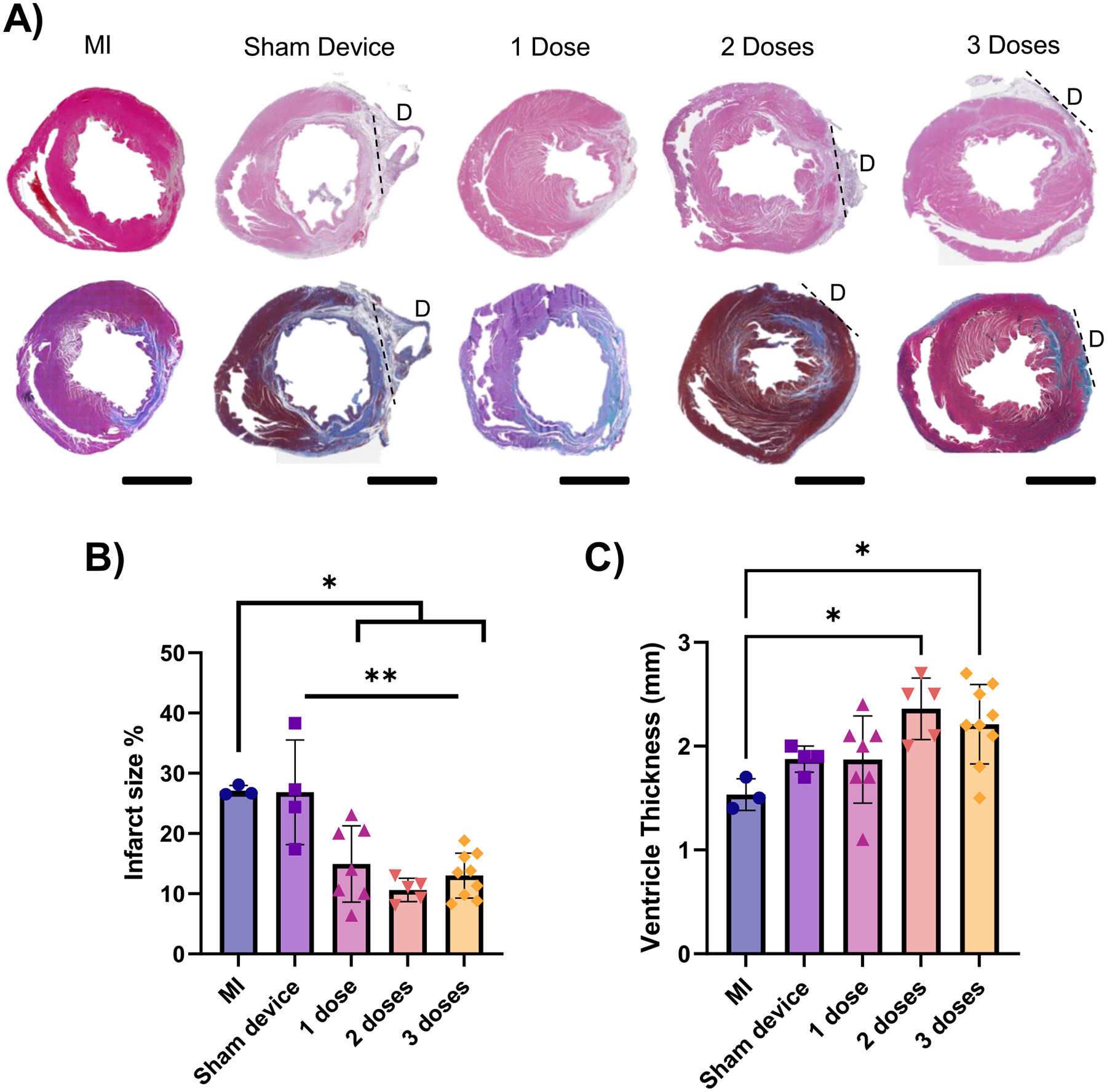
Histological assessment of infarct size and ventricle thickness. **A)** Representative H&E (top row) and Masson’s trichrome (bottom row) stained sections per group (scale bars are 2 mm; device positioning indicated by D). Infarct size **(B)** and ventricular thickness **(C)** as quantified by histology. *P < 0.05, **P < 0.005. Data are mean ± s.d. as analyzed by a one-way ANOVA with Tukey’s multiple comparisons tests. Individual animal values are shown as points in **B–C**.

Inspection of histological samples revealed prominent scar formation indicating an MI had occurred in all groups which is represented by hypochromatic staining in hematoxylin and eosin (H&E) sections and blue-stained collagen in Masson’s trichrome sections (Fig. 5A). A significant reduction of scarring (Fig. 5B) was observed when 1, 2, or 3 doses of FSTL1 are delivered when compared to the MI only and sham implant group. The double and triple FSTL1 dose regimens also led to a reduction of ventricular thinning compared to the MI only group (Fig. 5C). Since neither the 1 dose nor the sham implant group lead to a change in ventricular thickness, the double and triple dose regimens are likely mitigating the reduction of infarct extension post-MI solely biologically, via an FSTL1 specific pathway, or through a combined biomechanical mechanism, induced by FSTL1 and the mechanical benefit imparted by the epicardial implant.

###### Angiogenesis analysis:

**Fig. 6.**
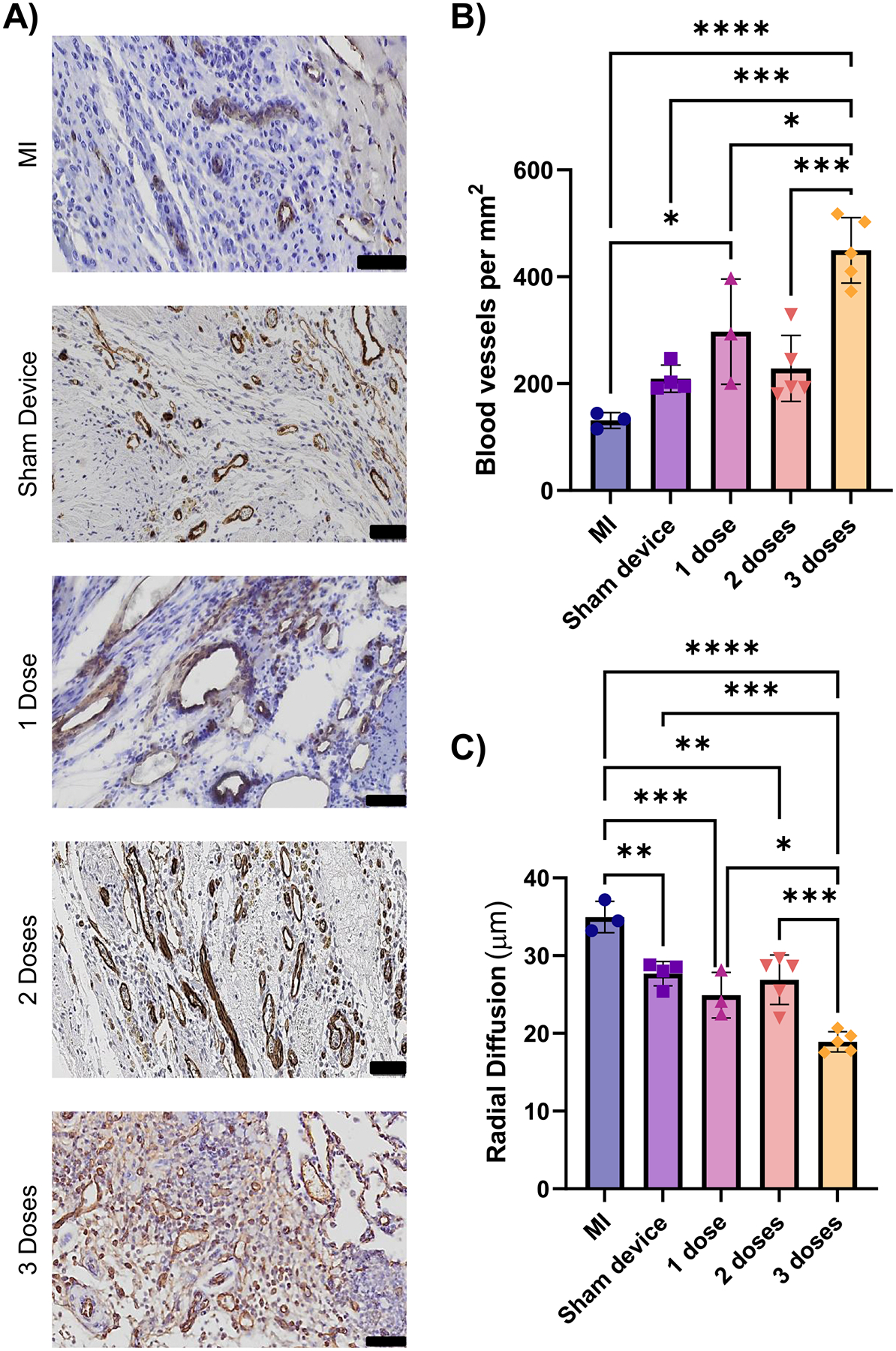
Histological assessment of angiogenesis. **A)** Representative CD31-stained sections per group (scale bar is 50 mm). Blood vessel number **(B)** and density **(C)** in the infarct zone as quantified by stereology. *P < 0.05, **P < 0.005, ***P < 0.0005, ****P < 0.0001. Data are mean ± s.d. (n = 3–5) as analyzed by a one-way ANOVA (Mixed model) with Tukey’s multiple comparisons post-test. Individual animal values are shown as points in **B–C**.

Three distinct zones were identified in all heart samples: the infarct zone (IZ) which was defined by a prominent collagen scar, the border zone (BZ), which surrounds the IZ and seems to be composed of hypertrophic cardiomyocytes, and the adjacent myocardium (AM) composed of morphologically healthy cardiomyocytes with prominent striations. Representative CD31-stained sections from the IZ, BZ and AM were used to quantify blood vessel number and radial diffusion, a metric of vessel-vessel proximity (Figs. 5, 6 and Fig. S6). As previously reported in a mouse model [27], we observed that exogenous FSTL1 induces a pro-angiogenic response in the IZ when compared to MI. Representative images of the IZ for all groups can be seen in Fig. 6A. The triple FSTL1 dose regimen significantly increased the number of blood vessels (Fig. 6B) and decreased neo-vessel-to-vessel distance (measured by radial diffusion, Fig. 6C) in the IZ when compared to all control and treatment groups.

An improvement in both blood vessel number and radial diffusion was observed with the single and double FSTL1 dose regimens when compared to the MI-only group. However, this improvement is comparable to the improvement observed with the sham implant group which, in the absence of FSTL1, could be attributed to mechanical reinforcement from the implant and the foreign body response observed (Fig. S4). Histological evaluation of the sham device group indicated hallmarks of late granulation tissue formation surrounding the reservoir. Further characterization of this interaction could include modulating the foreign body response to this indwelling implant through the incorporation of mechanical actuation [31,32]. Additionally, the inclusion of a group that delivers a single dose of FSTL1 using our epicardial implant, and not only a gelatin sponge, could help elucidate how differences in the foreign body response may influence this dosing regimen.

Improving the endogenous angiogenic response after MI can reduce scarring and adverse left ventricular remodeling [33]. In this study, the triple FSTL1 dose regime proved to be superior at inducing a pro-angiogenic response when compared to both the single and double dose regimens, despite comparable functional improvements. The time of delivery of the third FSTL1 dose may be important for generating an improvement in angiogenesis and is likely related to the interactions between FSTL1 and biological processes/cells characteristic of post-MI healing at day 21 [33,34]. Further studies are warranted to better understand how biodistribution and constant local changes in concentration with FSTL1 replenishment influences binding kinetics or saturation of further therapeutic effects. This enables further optimization of the time-specific therapeutic effect driven by multiple FSTL1 dosing regimens.

The epicardial system used for this study enables multi-dose delivery of bioagents *in vivo* without additional invasive procedures and has broad utility outside of this specific example. This delivery platform can be used to optimize the dosing regimen of FSTL1 and assess other bioagents for MI treatment and applications in other pathologies. It can also enable multi-agent therapeutic regimens. Incorporating more advanced features to our epicardial delivery platform such as continuous sensing for closed-loop bioagent delivery, may be required to maximize its therapeutic benefit for certain applications and increase its versatility which can ultimately contribute to its adoption in the clinic. This was the first study to deliver additional doses of FSTL1 in a non-invasive manner following implantation of an indwelling epicardial delivery platform in a rat model of MI.

### 2.4. Computational modeling to optimize design of future epicardial therapies

**Fig. 7.**
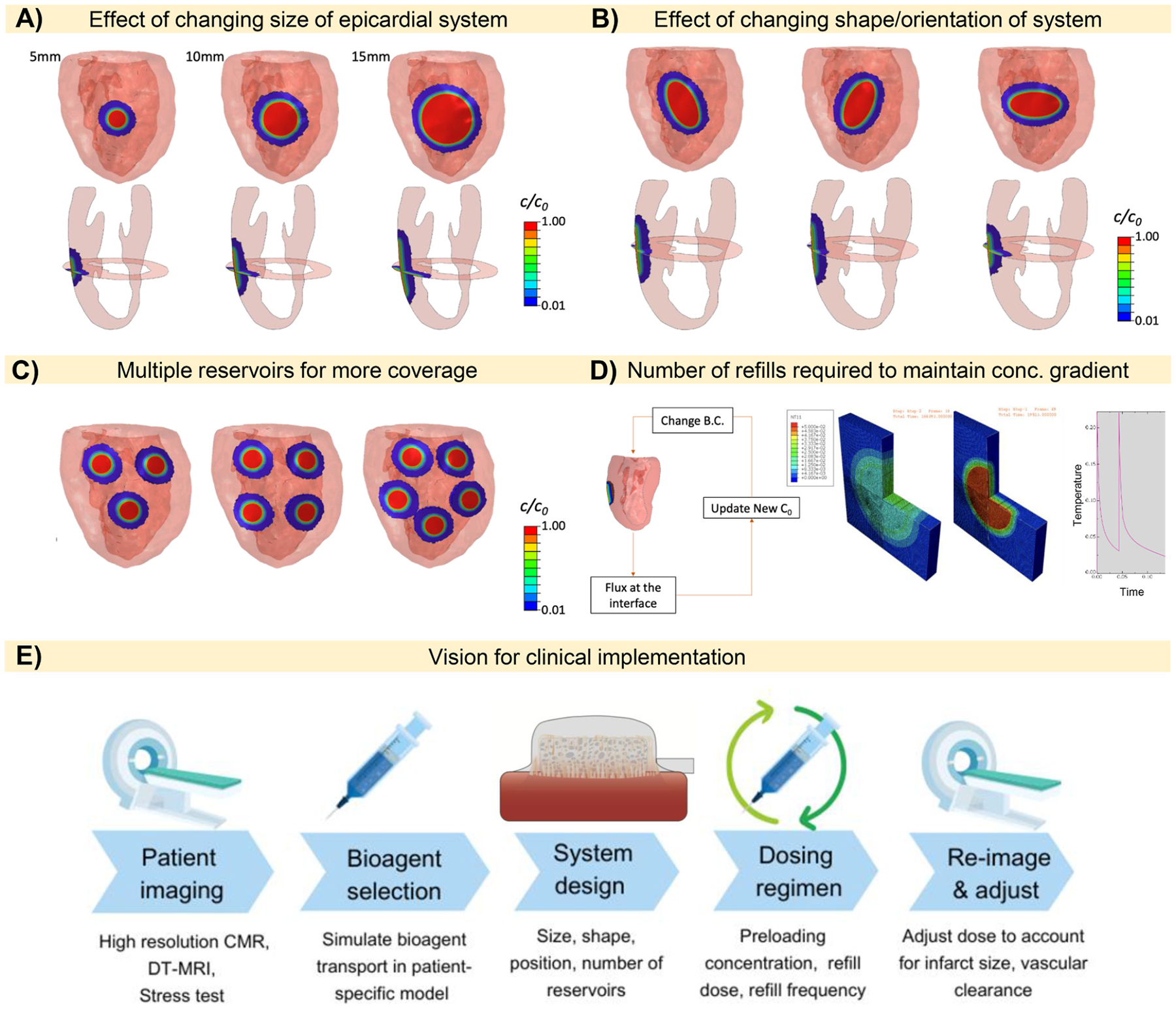
Computational modeling for per-patient planning. **A)** Effect of changing size of epicardial system. **B)** Effect of changing shape of epicardial system. **C)** Multiple reservoirs ensure more coverage of infarct and border zone. **D)** Modeling of refill can inform transmural transport of bioagent and inform refill frequency. **E)** Vision for clinical implementation including patient imaging, bioagent selection, system design, dosing regimen and ability to re-image and adjust.

In this work, we leveraged computational modeling of drug diffusion to characterize how microvascular clearance reduces drug concentration after local delivery, thereby motivating the need for multiple dosing to maintain therapeutic concentrations. With FSTL1 as a prototype therapeutic, we then used FE modeling to confirm the reservoir shape, placement, and dose to achieve therapeutic concentrations of FSTL1, and to guide design of the epicardial reservoir. Looking toward the future, we present a computational framework to further optimize system parameters such as reservoir size and shape, as well as dosing regimens. To demonstrate this, several FE simulations of diffusion into myocardial tissue were conducted, varying design parameters of the epicardial delivery system. Simulations varying the reservoir size showed that larger reservoir interfaces led to deeper and broader diffusion across all directions, including faster transmural penetration (Fig. 7A). When comparing different reservoir shapes, the diffusion profile initially conformed to the reservoir geometry but gradually became more aligned with myocardial fiber orientation over time (Fig. 7B). In the case of multiple reservoirs, closely spaced reservoirs resulted in earlier merging of diffusion fronts, with greater overall tissue coverage achieved as the number of reservoirs increased (Fig. 7C). Finally, the effect of refills was modeled by varying the boundary condition temporally, and could be used to optimize the dose and timing of delivered therapy (Fig. 7D). While the use of a uniform rodent MI model limited this study to a single reservoir design, we envision clinical implementation of this approach in which a patient undergoes clinical imaging to identify cardiac anatomy, myocardial structure, and infarct size and perfusion, and modeling is then used to optimize epicardial therapy dimensions, placement, and dosing. In this way, computational modeling can optimize epicardial therapy for each patient.

## 3. Materials and methods

### 3.1. Epicardial biomaterial system fabrication

The hemispherical shell of the epicardial biomaterial systems was manufactured as previously described [19]. The porous, tissue interfacing membrane was omitted, and instead a gelatin-based scaffold (Gelfoam^®^, or SurgiFoam^®^) was placed within the hemispherical shell, with direct exposure to the epicardium. Briefly, a thermoplastic polyurethane (TPU) sheet (HTM 8001-M polyether film, 0.003″ thick, American Polyfilm, Inc) was formed into a hemispherical reservoir using a vacuum thermal former (Yescom Dental Vacuum Former, Generic). The neck of the reservoir was heat bonded to a 10 cm, 3 Fr thermoplastic polyurethane catheter (Micro-Renathane 0.037″ × 0.023″, Braintree scientific) using a heat transfer machine (Heat Transfer Machine QXAi, Powerpress). Three holes were created around the perimeter of the polymer reservoir to guide suture placement during implantation. The assembled polyurethane implant components were sterilized using a low-temperature ethylene oxide cycle.

### 3.2. Franz Cell experiments

A Franz Cell study was conducted as previously described [35]. Gelatin biomaterial membranes (SurgiFoam^®^ 1972) were soak-loaded in PBS solution or methylene blue solution (500 μM; M9140, Sigma) and placed over the receptor chamber loaded with 8 mL PBS. Methylene blue solution (500 μL of 500 μM solution) was added to the donor chamber with membranes soak-loaded in PBS and the remaining un-absorbed methylene blue solution was added to the donor chamber with membranes soak-loaded with methylene blue solution. Samples of 330 μL were taken from the center of the Franz Cells after 5, 10, 15, 20, 25, 30, 35, 40, 45, 50, 55, 60, 90, 120, 150, 180, 240, 300, 360, and 1440 min. The donor chambers were reloaded with 500 μL of methylene blue solution (500 μM) and samples of 330 μL were taken from the center of the Franz Cells after 360 and 720 min (cumulative time of 1800 and 2160 min). Absorbance was measured at 665 ± 5 nm using a plate reader (HIDEX Sense Microplate Reader, Type 425–311). For the FSTL1 release study, gelatin sponges were soak loaded with 30 μL of FSTL1 (Aviscera Biosciences, 100 μg dissolved in 300 μL of PBS) were. Membranes were refilled with 30 μL of FSTL1 after 7 and 14 h. Samples were taken from the receiving baths at 7 h (1 dose), 14 h (2 doses) and 21 h (3 doses) prior to membrane refills. The concentration of FSTL1 delivered by the Gelatin biomaterial membranes at the pre-determined timepoints was determined using a human FSTL1 ELISA kit (Aviscera Bioscience, SK00372-01) and extrapolated from the standard curve. Absorbance was measured at 450 nm using a plate reader (HIDEX Sense Microplate Reader, Type 425-311).

### 3.3. Ex vivo testing of bioagent transport

The *ex vivo* setup and analysis were performed as previously described [21]. Two *ex vivo* assays were performed. In both assays, gelatin biomaterial cores (SurgiFoam^®^ 1972) were cut using a 4 mm biopsy punch, and soak-loaded in a methylene blue solution (1 mg mL–1; M9140, Sigma). In the first assay, the soak-loaded biomaterial cores were loaded into the device reservoir, sutured onto the epicardium of explanted Sprague-Dawley rat hearts (n = 2), and stored for 24 h at 37 °C with a water bath to maintain humidity. 20 h post-placement, cross-sectional images of tissue blocks were captured. Methylene blue intensity as a function of tissue penetration was quantified using ImageJ’s vertical Plot Profile function.

In the second assay, identically prepared soak-loaded biomaterial cores were placed on fresh chicken breast cubes (n = 2/group) to allow for bioagent proxy diffusion. For the 2-dose and 3-dose groups, 35 μL of the methylene blue solution was added to the gelatin scaffolds 1 h and 3 h after initial placement, respectively. Five hours post-placement, cross-sectional images of tissue blocks were captured. Methylene blue intensity as a function of tissue penetration was quantified using ImageJ’s vertical Plot Profile function.

### 3.4. Computational modeling

#### 3.4.1. Image-guided computational modeling of tissue transport

To characterize myocardial diffusion properties, a gadolinium-based contrast agent (Dotarem) was applied to a 4 × 4 cm^2^ swine myocardial tissue block via a reservoir affixed to the epicardium, and T1-mapping MRI was used to track the Gd diffusion profile over 14 h. Diffusion tensor MRI provided fiber orientation, and Mimics (Materialise NV, Leuven, Belgium) was used to reconstruct tissue geometry and map Gd concentration over time. Diffusion distances were measured along the fiber, sheetlet, and sheetlet-normal (transmural) directions, with a minimum detectable concentration determined to be 0.003 M based on T1 mapping. Using Fick’s second law (Equation (1)), direction-dependent diffusion coefficients were computed, showing initial variability before stabilizing around a fiber-to-cross-fiber diffusivity ratio of 0.58. The final diffusivities at 14 h —0.8979, and 0.4161 mm^2^/h in the fiber and sheetlet directions, respectively. The sheetlet-normal diffusivity was assumed to be the same as the sheetlet diffusivity, as these axes have similar microstructure and previous work has found diffusion along them to be comparable [36].

The diffusion FE model was built using Abaqus 2018 (Dassault Systèmes, Vélizy-Villacoublay, France) [37]. The reconstructed geometry was imported from Mimics and discretized in Abaqus/CAE with 102339 tetrahedron elements (DC3D4). Following a previous study [38], the post-processed DTI data was mapped to the FE model by assigning the orientation of the closest data point to the integration points of each element as its local orientation using MATLAB (The MathWorks, Inc., Natick, MA, USA). A thermal analogy as previously described [39] was used to simulate the diffusion process without microvascular clearance. The myocardium was assigned with density (1 × 10^−9^ *T*/*mm* [3]), specific heat capacity (1 mJ/t·K), and orthotropic thermal conductivities (for Gd, fiber - 2.49 × 10^−13^ *m K*, sheetlet and sheetlet-normal - 1.16 × 10^−13^ *mW*/*mm*·*K*), corresponding to the experimentally derived diffusivities mentioned above for Gd [40]. The Gd depot concentration (0.05 M) was converted to an equivalent temperature (0.0377 K) and applied as a boundary condition at the reservoir-epicardium interface, assuming a constant reservoir concentration. The simulation was run using the Abaqus/Implicit solver and predicted diffusion lengths for Gd were compared to MRI-based experimental measurements.

(Equation 1) $$\frac{\partial c}{\partial t}=\nabla \cdot \left(D\cdot \nabla c\right)$$

(where c is the concentration and ***D*** is the diffusivity.)

#### 3.4.2. Image-guided computational modeling of organ scale transport

Using a diffusion tensor MRI dataset of a healthy swine LV, a subject-specific FE model of the LV was developed following the same workflow as the tissue transport model. The calibrated diffusion coefficients were applied, with sheetlet and sheetlet-normal diffusivities set equal to reflect transverse isotropy of myocardial tissue. As shown in Fig. 6, one or more reservoirs were placed on the LV free wall, and diffusion was simulated for up to 10 days. A parameter study was conducted to assess the effects of reservoir size and orientation, including circular interfaces with radii of 5, 10, and 15 mm (Fig. 6A), and elliptical interfaces (15 × 7.5 mm) aligned in different orientations relative to epicardial fibers (Fig. 6B). Additional simulations with three to five circular reservoirs (radius 7.5 mm) evaluated the impact of reservoir patterning on diffusion coverage (Fig. 6C).

#### 3.4.3. Effect of vascular clearance on therapy residence in myocardial tissue

To model vascular clearance, we added a convection-based clearance rate to Fick’s law (Equation (2)), representing the rate at which molecules are cleared upon contact with microvessels. The vascular clearance depends on the permeability of the vessel wall (*P*_*mv*_), the microvascular volume fraction in the tissue (*ϕ*_*mv*_), and vessel diameter (*R*_*mv*_) [41]. The clearance rate (*k*) can be estimated using an empirical relationship (Equation (3)) between particle size and the ratio of permeability to diffusivity. For Gd, a small molecule contrast agent, the clearance rate was calculated to be 1.48 × 10^−5^ s ^−1^. For FSTL1, a larger biomolecule, the clearance rate was calculated to be 1.8 × 10 ^−5^s ^−1^

(Equation 2) $$\frac{\partial c}{\partial t}=\nabla \cdot \left(D\cdot \nabla c\right)-kc$$

(Equation 3) $$k=\frac{2}{R_{mv}}\frac{P_{mv}\phi_{mv}}{1-\phi_{mv}}$$

(where *R*_*mv*_ is vessel diameter, *ϕ*_*mv*_ is microvessel volume fraction, $P_{mv}=\frac{1}{D}$ is vessel wall permeability, D is the molecular diameter).

The clearance term was then implemented in an FE mass-diffusion model in Abaqus 2018 (Dassault Systèmes, Vélizy-Villacoublay, France) in which anisotropic diffusivity was assigned to the myocardium in the fiber and sheetlet and sheetlet-normal directions. For Gd, the aforementioned experimentally-derived diffusivities were used. For FSTL1, diffusivity along the fiber was estimated by the Stokes-Einstein Girier-Wirtz Estimation (SEGWE) [40] and scaled according to the experimental results for Gd to additionally estimate the sheetlet and sheetlet normal diffusivities. The FSTL1 depot concentration (9.15e-9 M) was applied as a boundary condiiton at the reservoir-epicardium interface, assuming a constant reservoir concentration. The simulation was run using the Abaqus/Implicit solver and predicted diffusion lengths for FSTL1 were used to support epicardial reservoir design. Important limitations of the FSTL1 simulations include that the SEGWE does not incorporate electrical charge, and that the relationship between diffusivities along the fiber and normal to or along the sheetlet was not derived experimentally. However, given the nearly neutral pH of the heart tissue (7.1–7.2) and inability to extend the diffusion tensor MRI approach to FSTL1 because it is not radiopaque, we believe our methods offered sufficient resolution to guide the design of the reservoir [42].

### 3.5. Animal study

Animal procedures were reviewed and approved according to ethical regulations by the Institutional Animal Care and Use Committees at Brigham and Women’s Hospital and the Massachusetts Institute of Technology. For this 28-day study, female Sprague Dawley rats (225–250 g) were assigned to the following groups: 1) sham implant (epicardial implant with PBS-loaded gelatin sponge), 2) single FSTL1 dose (via FSTL1-loaded gelatin sponge), 3) 2 doses of FSTL1 (FSTL1-loaded gelatin sponge and 1 refill via epicardial implant), and 4) 3 doses of FSTL1 (FSTL1-loaded gelatin sponge and 2 refills via epicardial implant). For comparison, we report functional and histological data from rats that received no treatment (MI only) measured using the same methods and personnel and reported previously [19].

#### 3.5.1. Surgical procedure

MI creation, and implantation of the gelatin sponge and/or epicardial implant were performed as previously described [19]. Briefly, rats that received a system implantation were anesthetized (1–3% isoflurane in O_2_), shaved at the dorsal (between the shoulder blades) and ventral (left side of the chest) surgical sites, and injected with a regional nerve blocker (lidocaine/bupivacaine) and a pre-operative analgesic (buprenorphine, 0.05 mg/kg) subcutaneously. Endotracheal intubation was performed, and subcutaneous incisions were made at the dorsal and ventral surgical sites. No dorsal incision was performed in groups where no epicardial implant was implanted. The system refill line was tunneled subcutaneously from the dorsal to the ventral incision before MI creation. A thoracotomy was performed between the fourth and fifth inter-costal space and the pericardium was removed using forceps. A MI was created by permanently ligating the LAD coronary artery with a suture (6-0 or 7-0 prolene) approximately one third of the way from the base to the apex of the heart. After MI induction, a gelatin sponge was either directly sutured onto the heart (single suture, 7-0 prolene) or placed inside the system shell for subsequent attachment. The reservoir was sutured (7-0 prolene) to the surface of the heart at 3 previously marked points. After gelatin sponge or system attachment, the thoracotomy and ventral incision were closed (layer-by-layer, 4-0 vicryl). In rats with an epicardial system, a vascular access button, or self-sealing port (VAB62BS/22, Instech Laboratories), was then connected to the end of the system refill line and placed subcutaneously between the shoulder blades of the rat. The port was secured to the underlying fascia using at least two interrupted sutures (4-0 vicryl) prior to skin closure. Animals were ventilated with 100% oxygen on a heated pad until autonomous breathing was regained. 3 ml of warm saline was administered subcutaneously, and buprenorphine (0.05 mg/kg in 50 μl IP) was given every 12 h for three days post-operatively.

#### 3.5.2. FSTL1 delivery

The initial dose of FSTL1 was delivered to the treatment groups through a FSTL1-loaded gelatin sponge. A sterile 4-mm biopsy punch was used to cut a core of commercially available sterile gelatin sponge (Gelfoam^®^, or SurgiFoam^®^) inside a biological hood. The core was soaked for at least 30 min prior to implantation in a solution containing 10 μg of FSTL1 (Aviscera Bioscience, suspended in 30 or 100 μl of sterile PBS). Forceps were used to gently tap the gelatin sponge core to promote absorption of the FSTL1 solution. FSTL1-loaded gelatin sponges were secured directly onto the infarct region of rats in the single dose group (without the epicardial system) or placed inside the system hemispherical shell prior to suturing the system to the heart in the double and triple dose groups. A PBS solution was used to soak the gelatin sponge cores placed inside the systems implanted into the sham implant group.

The double and triple dose groups received either 1 or 2 FSTL1 refills, respectively. The first FSTL1 refill (second dose) occurred at day 14 post-MI while the second refill (third dose) occurred at day 21 post-MI (Fig. 1C). For each refill, the rat was anesthetized (1–3% isoflurane in O_2_) and negative pressure was applied to clear the refill line of the system using an empty syringe connected to the self-sealing port. Then, 3 μg of FSTL1 (Aviscera Biosciences, suspended in 30 μl of sterile PBS) were injected through the port into the refill line followed by 20 μl of PBS to clear the refill line from any FSTL1 and ensure the FSTL1 reached the epicardial depot.

#### 3.5.3. Echocardiography

Echocardiography was conducted at days 1, 7 and 28 for all groups (Fig. 1c). Data was acquired using either a Vevo 2100 or 3100 Ultra-sound machine and a MS200 transducer probe (9–18Mhz). End systolic volumes (ESV), EDV and EF measurements were obtained from B-mode, parasternal long axis images. FS measurements were obtained using an M-Mode parasternal short axis view of the heart, at mid ventricular level, with papillary muscles evident. Data was analyzed using the Visualsonics software (VevoLAB). The imaging datasets were analyzed and reviewed by two experienced ultrasound readers blinded to the study groups.

#### 3.5.4. Hemodynamic evaluation

At day 28, invasive hemodynamic recordings were performed as previously described [19,39]. Briefly, a sternotomy was performed, a 5-0 silk suture snare was placed around the inferior vena cava (IVC), and a pressure-volume catheter (Millar SPR-838) was inserted into the LV via an apical stick. After baseline recordings, pressure-volume relationships over a range of preloads were recorded as the IVC was temporarily occluded using the placed snare. LabChart Pro 8 (AD Instruments) was used for all pressure-volume data analysis. The volume signal was calibrated by inputting ESV and EDV obtained from echocardiography measurements. EDPVRs were obtained by performing an exponential curve fit of ~10 pressure-volume loops recorded during an IVC occlusion with LabChart’s pressure-volume loop module (*Ped* = a*exp (b*Ved), where *Ped* and *Ved* respectively correspond to end diastolic pressure (mmHg) and volume (*μ*l), and *a* and *b* are fit parameters). The slope (*b*) of 1–4 EDPVRs was averaged to derive each animal’s chamber stiffness value.

#### 3.5.5. Histology

At the terminal point of the study, rats were anesthetized, and their hearts were arrested in diastole by direct injection of 2–3 ml of 1% *KCl* solution and fixed in a 10% neutral buffered formalin solution for 24 h. Explanted hearts were sliced into three transverse sections (apex, mid-ventricle, and base). Heart slices were placed into cassettes and processed using a tissue processor through increasing grades of ethanol to paraffin wax. Samples were then wax embedded and sectioned into 5 μm slices using a microtome and mounted onto glass slides for histochemistry and immunohistochemistry. H&E and Masson’s trichrome staining was performed using previously established protocols [40]. CD31 immunohistochemistry was performed to stain blood vessels. Briefly, samples were dewaxed and rehydrated through decreasing grades of ethanol, and heat mediated antigen retrieval was performed using sodium citrate with 0.01% tween. Next samples were blocked using peroxidase, incubated with primary CD31 antibody for 1 h at room temperature (ab182981), and stained with 3,3 diaminobenzidine (DAB) using the Abcam staining kit (ab236469).

#### 3.5.6. Stereology

All quantitative analysis was performed using ImageJ from a blinded counter. For all measurements, test fields were gathered and a point grid with an adjusted number of test points was projected onto each test field. H&E and Masson’s trichrome stained samples were used to measure left ventricular thickness. A perpendicular line was drawn from each point where the grid intersected the inner aspect of the LV wall to the outer aspect and the length was measured using the measure function with a defined scale bar. Masson’s trichrome stained hearts were used to determine the extent of myocardial scarring. The area fraction of scar tissue was estimated by applying a grid to the tissue and counting the number points overlaying scar tissue on the LV which was divided by the number of points overlying the LV and expressed as a percentage. Less dense fibrous capsule scar was removed during this measurement. Stereological measurements of blood vessels were performed as previously shown [40,41]. Briefly, ten non-overlapping images were taken from the infarct zone, border zone, and adjacent myocardium. The number of blood vessels was quantified using a systematic randomized sampling approach with an unbiased counting frame. The number of blood vessels per area was determined and the length density (2* the number per area) and radial diffusion distance were determined (1/√π * Length density) for each heart. For each type of analysis, 2–4 sections were analyzed from different regions of the heart.

### 3.6. Statistics

Statistical analysis was performed using GraphPad Prism. All graphs are expressed as a mean (± standard deviation) with means for individual animals shown as points. Normality tests were performed using a Shapiro-Wilks test and either a one-way ANOVA with post hoc Tukey’s test, or a 2-way ANOVA (Mixed model) with post Dunnet’s test were performed to test for significance. Differences between groups were considered statistically significant to each other if p < 0.05.

## 4. Conclusions

In this study, we propose a new epicardial reservoir system which allows direct biomaterial integration with host tissue and provides a sustained local concentration of bioagent to enhance myocardial transport. We employ experimental and computational simulations to characterize bioagent transport from the biomaterial into tissue, using drug analogs (methylene blue, Gd). We demonstrate the ability to simulate the effect of biomaterial choice (permeability, retention of bioagent), system design (size, shape, orientation), tissue properties (myocyte orientation, vascularity of tissue) and bioagent (hydrodynamic radius, number of refills).

In a rat MI model, we demonstrate functional effects of this epicardial reservoir system. We deliver multiple FSTL1 doses non-invasively and characterized the therapeutic benefit of single, double, and triple dose regimens without the effects of additional surgical interventions. We demonstrate the beneficial effects of epicardial FSTL1 delivery previously reported in mouse models can be recapitulated with a rat model of MI and our epicardial system. A single dose of FSTL1 yields a higher EF and/or FS in FSTL1 treated groups in comparison to MI controls. By day 28 after MI, 3 doses of FSTL1 lead to the greatest increase in EF and FS in comparison to MI controls. FSTL1 decreases chamber stiffness after 2 doses in a dose-dependent manner. A reduction in infarct size is observed after delivering FSTL1. Multidose FSTL1 regimens lead to increased ventricular wall thickness, blood vessel number and density in the infarct zone in comparison to MI controls, with 3 doses resulting in significantly improved angiogenesis in comparison to all other groups.

Finally, we posit that patient-specific design of the system and dosing regimen with longitudinal monitoring and adjustment could improve cardiac recovery post-MI. We have previously scaled up similar replenishable implants for clinical translation [42]. Our delivery platform may play an essential role in facile assessment of timed dosing regimens for various bioagents or bioagent combinations.

## Supplementary Material

Supplemental data Appendix A

## Data Availability

Data will be made available on request.

## Appendix A. Supplementary data

Supplementary data to this article can be found online at https://doi.org/10.1016/j.biomaterials.2026.124087.
