# Supplementary material for: Multimodal characterization of sustained bioagent release from an epicardial depot for long-term biomaterial incorporation
Source: Biomaterials. Author manuscript; Available in PMC 2026 Aug 1. (PMC13263067; doi:10.1016/j.biomaterials.2026.124087)
Supplement: Supplemental data Appendix A [file NIHMS2180582-supplement-Supplemental_data_Appendix_A.docx]

Supporting Information

**Multimodal characterization of sustained bioagent release from an epicardial depot for long-term biomaterial incorporation**

*Claudia E. Varela^1,2^*, David S. Monahan^1,3^*, Yiling Fan^4^*, Shahrin Islam^4^, Jane Tunde Kelleher^1,2^, William Whyte^1^, Jean Bonnemain^1,5^, Souen Ngoy^6^, Sudeshna Fisch^6^, Eimear Wallace^7^, William Ronan^7^, Rachel Beatty^8^, Eimear B. Dolan^3,7^, Christopher T. Nguyen^9^, Garry P. Duffy**^3^, Ellen T. Roche**^1-4^*

1. Institute for Medical Engineering and Science, Massachusetts Institute of Technology, Cambridge, MA, USA.

2. Harvard-MIT Program in Health Sciences and Technology, Cambridge, MA, USA.

3. CÚRAM Research Ireland Centre for Medical Devices, College of Medicine Nursing and Health Sciences, University of Galway, Galway, Ireland.

4. Department of Mechanical Engineering, Massachusetts Institute of Technology, Cambridge, MA, USA.

5. Department of Adult Intensive Care Medicine, Lausanne University Hospital and University of Lausanne, Lausanne, Switzerland

6. Department of Medicine, Brigham & Women's Hospital and Harvard Medical School, Boston, MA, USA

7. Biomedical Engineering, College of Engineering and Science, University of Galway, Ireland.

8. Anatomy Department, College of Medicine, Nursing and Health Sciences, University of Galway, Ireland.

9. Cardiovascular Institute, Cleveland Clinic, OH, USA

* These authors contributed equally and are co-first authors

** These authors contributed equally and are co-senior authors

**Figure S1. Amount of FSTL1 released over 7, 14 and 21 hours when additional doses are supplied at 7 and 14 hours using a Franz cell set up.** Data are mean ± s.d. (n =3). Individual values are overlayed as points.

**
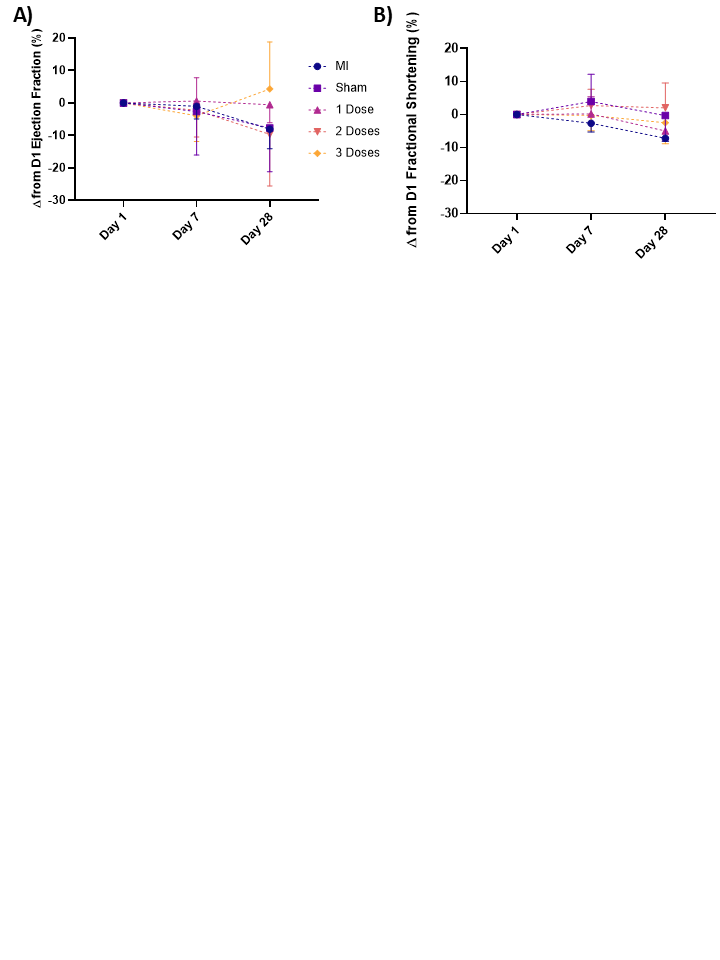
**

**Figure S2. Change in ejection fraction and fractional shortening with respect to Day 1.** Data are mean ± s.d. (n =3-5). Individual values are overlayed as points.

**Figure S3. End diastolic volume as assessed by echocardiography.** Data are mean ± s.d. (n =4–8) as analyzed by a one-way ANOVA (Mixed model) with Tukey’s multiple comparisons post-test. Individual values are overlayed as points.

**
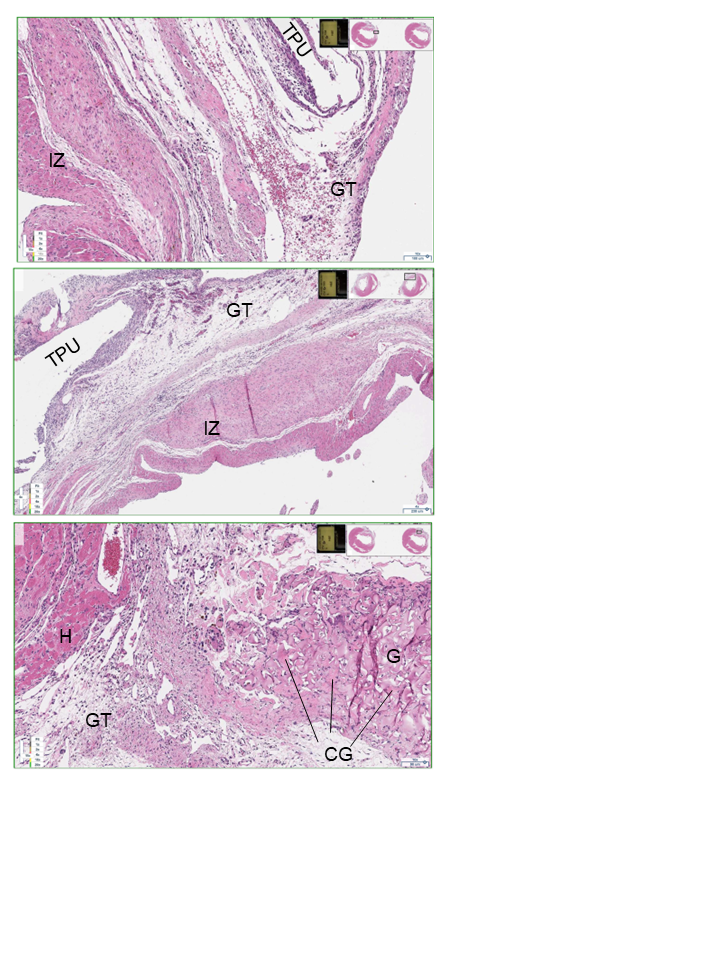
**

238μm

100μm

100μm

**Figure S4. Sham device histology to assess reservoir/gelatin/tissue interface.** Representative subjects showcasing late granulation tissue (GT) near the expected polymer reservoir location (TPU). Bottom image shows gelatin scaffold is starting to become integrated with the tissue over the timeframe of the study. G:Gelatin sponge, CG: Cells in Gelatin sponge likely replacing it, H: heart tissue, GT: granulation tissue, IZ: infarct zone.

**
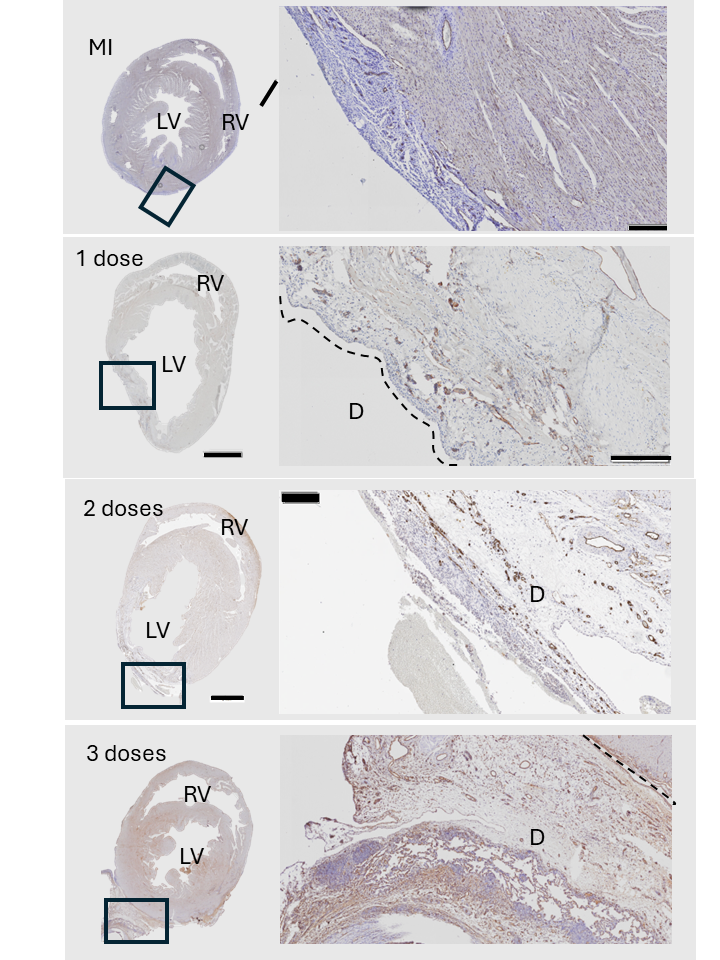
**

**Figure S5. Additional histological assessment of angiogenesis. A)** Representative CD31-stained sections of experimental groups (scale bar is 2 mm and 200 μm). Dashed line represents gelatin sponge or polymer reservoir interface with epicaridal surface. D: Assumed reservoir device location, RV: right ventricle, LV: left ventricle.


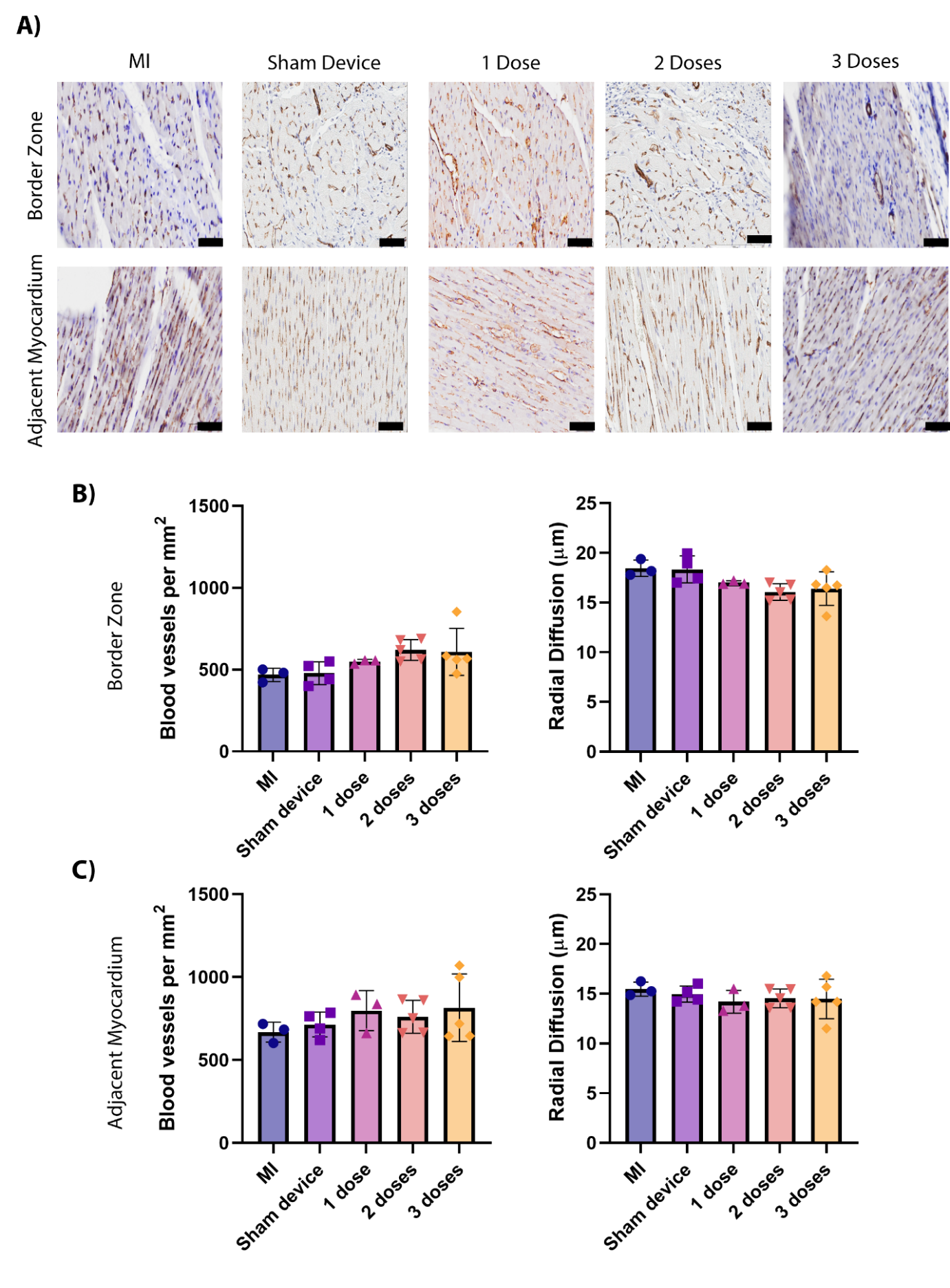


**Figure S6. Additional histological assessment of angiogenesis.** **A)** Representative CD31-stained sections of border zone and adjacent myocardium per group (scale bar is 50 μm). Blood vessel number and density in the border zone **(B)** and adjacent myocardium **(C)** as quantified by stereology. * P < 0.05, ** P < 0.005, *** P < 0.0005, ****P < 0.0001. Data are mean ± s.d. (n = 3–5) as analyzed by a one-way ANOVA (Mixed model) with Tukey’s multiple comparisons post-test. Individual values are overlayed as points in **B-C**
